# Investigating the Acceptance of Video Consultation by Patients in Rural Primary Care: Empirical Comparison of Preusers and Actual Users

**DOI:** 10.2196/20813

**Published:** 2020-10-22

**Authors:** Marius Mueller, Michael Knop, Bjoern Niehaves, Charles Christian Adarkwah

**Affiliations:** 1 Chair of Information Systems University of Siegen Siegen Germany; 2 Department of General Practice and Family Medicine Philipps-University Marburg Germany; 3 Department of Health Services Research CAPHRI Care and Public Health Research Institute Maastricht University Maastricht Netherlands

**Keywords:** video consultation, technology acceptance, digital health care technology, primary care, rural areas, telemedicine, behavioral intention, eHealth, teleconsultation, electronic consultation, general practitioners

## Abstract

**Background:**

The ongoing digitalization in health care is enabling patients to receive treatment via telemedical technologies, such as video consultation (VC), which are increasingly being used by general practitioners. Rural areas in particular exhibit a rapidly aging population, with an increase in associated health issues, whereas the level of attraction for working in those regions is decreasing for young physicians. Integrating telemedical approaches in treating patients can help lessen the professional workload and counteract the trend toward the spatial undersupply in many countries. As a result, an increasing number of patients are being confronted with digital treatment and new forms of care delivery. These novel ways of care engender interactions with patients and their private lives in unprecedented ways, calling for studies that incorporate patient needs, expectations, and behavior into the design and application of telemedical technology within the field of primary care.

**Objective:**

This study aims to unveil and compare the acceptance-promoting factors of patients without (preusers) and with experiences (actual users) in using VC in a primary care setting and to provide implications for the design, theory, and use of VC.

**Methods:**

In total, 20 semistructured interviews were conducted with patients in 2 rural primary care practices to identify and analyze patient needs, perceptions, and experiences that facilitate the acceptance of VC technology and adoption behavior. Both preusers and actual users of VC were engaged, allowing for an empirical comparison. For data analysis, a procedure was followed based on open, axial, and selective coding.

**Results:**

The study delivers factors and respective subdimensions that foster the perceptions of patients toward VC in rural primary care. Factors cover attitudes and expectations toward the use of VC, the patient-physician relationship and its impact on technology assessment and use, patients’ rights and obligations that emerge with the introduction of VC in primary care, and the influence of social norms on the use of VC and vice versa. With regard to these factors, the results indicate differences between preusers and actual users of VC, which imply ways of designing and implementing VC concerning the respective user group. Actual users attach higher importance to the perceived benefits of VC and their responsibility to use it appropriately, which might be rooted in the technological intervention they experienced. On the contrary, preusers valued the opinions and expectations of their peers.

**Conclusions:**

The way the limitations and potential of VC are perceived varies across patients. When practicing VC in primary care, different aspects should be considered when dealing with preusers, such as maintaining a physical interaction with the physician or incorporating social cues. Once the digital intervention takes place, patients tend to value benefits such as flexibility and effectiveness over potential concerns.

## Introduction

### Background

In many countries, health care systems are facing increasing challenges that are obliging care providers as well as consumers to adapt. In many rural regions, a shortage of physicians, especially general practitioners (GPs), is obvious and will dramatically increase in the near future [1-3]. A smaller number of GPs will have to take care of a larger number of patients because of demographic changes and an aging population, and catchment areas will increase [4]. Furthermore, GPs—especially in rural areas—have problems finding successors for their practices [5,6]. As a result, imbalances, disparities, and inequitable distributions of care occur, which threaten the comprehensive provision of care and the maintenance of population-wide health [7,8]. The short-term availability of care and medical expertise to which patients are accustomed is at risk. Accordingly, the patients’ readiness to change the process of care delivery represents a major governmental as well as scientific issue.

The digitalization of health care processes and treatments over the last two decades represents a promising measure to counteract these issues. A large number of digital technologies have been applied within different medical domains to bridge the emergent gaps in patient treatment, ranging from preventive tools to rehabilitation support systems [9]. For instance, technological advancements in care occur in the form of digitalized patient-physician communication and consultation via web-based video consultation (VC) [10], which enables, for example, remote examinations [11,12], virtual visits at patients’ homes [13], and the involvement of relatives and caregivers [14]. Further applications cover the remote collection of patient data through user input or body-worn sensors measuring vital parameters [15,16]; digital prescriptions [17] and web-based scheduling [18]; web-based provision of information on diseases, symptoms, and treatments [19]; and telemonitoring of patients [20].

Clearly, the beneficial implementation, evaluation, and continuous use of health care technologies are vital [21]. A crucial factor for this is the users’ acceptance of the technology in play [22,23]. Accordingly, the investigation of factors determining the acceptance of telemedical technology by patients in rural areas represents a major scientific task. Technology acceptance by patients has been subject to several studies [24-26], using models such as the Technology Acceptance Model (TAM) [27,28], the Unified Theory of Acceptance and Use of Technology (UTAUT) [29], or models based on the Theory of Planned Behavior (TPB) [30,31]. However, in the case of telemedicine, these models deliver varying results [23,32], using a wide spectrum of variables without preselection [33,34]. Furthermore, the proposed models often neglect contextual factors and have a narrow view of complex phenomena [35]. Prior models might deliver results that have low explanatory power with regard to primary care settings. Consequently, this study takes an exploratory approach to shed further light on the acceptance of VC as a prominent representative of telemedicine in primary care.

### Objectives

Previous studies have looked at telemedical support, for example, in the form of mobile apps, in the case of specialized care and for specific indications, such as palliative medicine [36] or stroke care [13,37]. When looking at primary care, VC represents a telemedical solution that has already been used widely by GPs and specialists to offer innovative ways of patient care and to cope with increasing challenges. A few studies have investigated how patients and medical professionals experience the use of VC systems in primary care [38-42]. Although these studies delivered first insights into the use and acceptance of VC by patients, the focus was predominantly on the convenience and benefits of VC [39,40], and an in-depth study seeking to unveil the social, personal, technical, environmental, and organizational factors affecting the use of VC in primary care remains to be done. In addition, the samples involved do not account for the vast majority of patients who have not yet encountered VC for treatment and are thus still in the process of forming behavioral intentions and attitudes toward VC in primary care.

Thus, the objectives of this study are (1) to empirically identify factors that drive patient evaluation, acceptance, and utilization of VC technologies, using research on patients with and without experience in using such a system within rural primary care; and (2) to contrast these 2 populations to expose the differences and commonalities that are potentially rooted in digital interventions. On the basis of these findings, implications can be drawn for design, application, and theory. This paper contributes to understanding what is important to patients in their roles as preusers as well as actual users of VC. The paper focuses on primary care setting because it affects a majority of citizens, from chronic patients who are obliged to interact frequently with their physicians to patients with nonsevere and acute diseases that render visits and consultations occasional. As the supply situation mentioned earlier reveals rural areas are threatened by a shortage of GPs, this study investigates patients and practices in a representative rural area in Germany.

## Methods

### Study Design

We conducted a qualitative study, as part of a regional project agenda, empirically investigating the digitization of primary care practices and health care processes in the German setting, focusing in particular on the specific conditions in rural areas. With regard to our study design, we seek to empirically explore and identify factors that shape patients’ perceptions, evaluations, adoption, and continuous use of VC in the primary care setting, focusing on the patient perspective. We conducted semistructured interviews with patients with and without experience in using VC, which allows for a comparison of these 2 patient cohorts and unveil differences and commonalities in what is important to the patients. We draw upon the notion of *preusers*, who are “[...] individuals and groups who do not have well-developed notions of how digital technologies fit into or affect their lives” [31]. As telemedical solutions such as VC are not widely used in health care [43,44], this user group represents a majority of patients. On the other hand, some primary care practices have already adopted VC systems for patient treatment. Accordingly, the population of patients who have actually encountered telemedicine is growing, forming a group of actual users who potentially pursue different norms, beliefs, and behaviors. Hence, we engage both preusers and actual users for 3 reasons. First, the intention to use a system is a major predictor of actual use [27] and is formed beforehand based on expectations and, in many cases, lack of actual experience. Therefore, it is useful to interrogate preusers to shed light on the emergence of behavioral intentions. Second, to establish fair and equitable access to care, it is important to include all patients who have already used or potentially will use VC for treatment. This includes patients with a lack of technical affinity or willingness to participate in VC; hence, they might remain preusers. It is important to determine what drives or hinders these patients from using VC. Finally, from a provider’s perspective, the economic success of implementing telemedicine seems important. Here, achieving a critical mass of users is crucial, calling for comprehensive technology acceptance to transform preusers into actual users.

### Data Collection

All 158 GP practices in the region of Siegen-Wittgenstein, Germany, were contacted and asked for their experience with VCs. Of these practices, only 2 GPs stated that they had intensive experience with this method of treatment. These 2 GP practices included VCs in their regular office hours, that is, patients can opt for a VC or a face-to-face consultation (FTFC). For a VC, patients have to register and book an appointment through the website of the GP practice. Afterward, a link is sent to the patient via a text message and email, specifying the date and time of appointment. Finally, the patient needs to click on the link to enter the conference room. The GP immediately gets a notification when a patient is online and can start the VC. Patients were offered VC use instead of FTFC. All patients who registered for a VC were consecutively asked to participate in the study to have a representative sample of practice attendees. Owing to the COVID-19 pandemic, there was great interest in VCs among patients, and only one patient in each practice refused to take part. In rural GP practices in Germany, the number of patients registered is higher on average than in practices in urban regions. Furthermore, the proportion of older patients is somewhat greater. This is also the case for the practices participating in this study.

We conducted 20 semistructured interviews, drawing samples from these 2 primary care practices. Interviews were carried out in 2 phases. In phase 1, we conducted 10 interview sessions (sample A) at the first site with patients who did not have any experience with VCs. Thus, sample A represents the preuser group. In phase 2, we conducted 10 additional interviews with participants from the second practice (sample B) who had already used a VC system to consult their physician. As 4 of these interviews took place digitally because of the COVID-19 pandemic, interviewees were asked to evaluate their VC experience despite the acute circumstances (eg, restrictions on personal contact) if possible. In doing so, we aimed to collect coherent data. Sample B forms the actual user group. Both samples were recruited via 2 GP practices, as mentioned earlier, who reached out to suitable patients willing to participate in the project, thus allowing a convenient (sample A) and purposeful (sample B) sampling approach [45]. The sample yielded a total of 22 interviewees, 9 women and 13 men, with an average age of 51.2 years (SD 19.2). Interviews took 26 min on average and were conducted from August 2019 to April 2020. The comprehensive sample and interview process characteristics are illustrated in Multimedia Appendix 1. The samples thus consisted of patients with different education levels, age, gender, and health status. We tried to recruit samples that (1) adequately represent the common client base of rural primary care practices in the investigated region and (2), in the case of sample B, can be seen as recurrent users of telemedicine according to the physicians and self-disclosure.

The interview guideline covered 5 questions groups seeking to unveil different factors explaining the patients’ attitudes toward VC and adjacent telemedical solutions. Questions covered patient, social, environmental, and organizational as well as technical and interaction factors, building upon the classification by Or and Karsh [33]. The guideline was adapted between the interview phases to reflect on the varying level of experience with VC between samples. However, we did not change the guideline in between interviews of the same sample, thus avoiding the possibility that the interviewees’ statements could influence each other. In doing so, we aimed for unbiased data because the attitudes and perceptions under investigation are highly individual. Both interview guidelines are presented in Multimedia Appendix 2. In the case of sample A, a technical scenario was presented to the interviewees at the beginning of each interview to allow for a common understanding of telemedical treatments. The scenario involved 2 components: first, a live VC with the GP about distance, for example, from home; and second, mobile sensory equipment that enables patients to measure and transfer vital parameters (eg, blood pressure) on their own. In the case of sample B, for the sake of comparability, the application of sensor equipment besides the experienced VC was introduced to the interviewees at the end of each interview session. Here, we additionally asked for the patients’ perception of the usefulness and applicability of the sensory equipment in future treatments.

The interviews were conducted in German by 2 members of the research group, audio recorded, and transcribed nonverbatim, leaving out pauses and off-topic exchanges of words. Before each interview, the interviewees signed an informed consent, inter alia briefing them about voluntariness, the anonymization and partial publication of data, and their right to withdraw their study participation. For the sake of analysis, comparability with literature, and reporting, the transcripts were translated into English. The study was approved by the data protection commissioner of the University of Siegen.

### Data Analysis

To reflect our data collection procedure, we followed a two-phased data analysis approach consisting of an inductive phase, analyzing data from sample A, and a subsequent deductive phase, analyzing data from sample B. Here, the results from phase 1 are used for analysis in phase 2. This procedure allows factors to persist, but also to change, complement, or substitute each other or be canceled out entirely. In this way, differences as well as commonalities between preusers and actual users become visible.

In the first phase, we analyzed the data gathered from sample A inductively to identify and comprehensibly define the first set of relevant factors associated with preusers. We followed a three-step approach [46]. First, 2 authors coded the interview data independently. The approach proposed by Strauss and Corbin [47], consisting of open and axial coding, was followed. Selective codes were formed by subsuming redundant and/or related codes into superordinate categories that represent factors with regard to the patients’ attitudes toward and their acceptance of the technologies involved. Second, the 2 coding authors discussed their individual categorizations, merging codes with similar reasoning and formulation, and resolved disagreements. Consequently, some of the standalone codes were subsumed under others because they represented a particular facet of the resulting factor. This procedure led to a first categorization scheme consisting of 3 groups, which involve 7 subsumed factors, and 1 standalone group, which forms a factor by itself. Finally, based on the elaborate scheme, each involved researcher recoded the data, assigning the 8 factors to the interviewees’ statements. After that, a final discussion on categories, their dimensions and facets, and factor-to-data assignments was carried out.

In the second phase, we analyzed the data collected from sample B in a deductive manner. Here, the final coding scheme from the first phase was applied as the initial template to code the remaining data. Again, the data were coded in 3 steps as in phase 1. First, the authors independently assigned identified codes to the data, allowing new codes to emerge and existing codes to be redefined. Statements that did not fit in the coding scheme were again coded following the inductive approach described earlier (open, axial, and selective coding). This led to a new factor dealing with patient responsibilities and obligations, which included novel insights with regard to the actual user group. Second, step 2 was carried out analogous to the first phase, leading to a new merged categorization that comprised the extended 4 factor groups, followed by, finally, a recoding of the data by both members of the research group. Before recoding, the raters agreed upon the data segments to which codes were assigned. To check for interrater reliability of the coding performed, we calculated Cohen kappa [48] after the final recoding of all the data was done (see step 3 during data analysis). The resulting value of 0.75 indicates a substantial agreement in coding and, thus, sufficient reliability [49].

Multimedia Appendix 3 shows the quantity of interview coding that relates to the factors after recoding of the data and the number of interviews that involve the respective factor. Both samples are presented individually and complemented by total numbers.

## Results

### Overview

In total, 4 different design and application relevant factor groups (attitudes and expectations, human interaction, rights and obligations, and social factors), each with their respective subdimensions, emerged from samples A and B. Although the context and connotations of specific factors varied between our 2 samples, we explored interesting commonalities and differences. The presented findings come from the experiences of patients with no experience in digital or video-based treatment (sample A) and those who have already experienced VCs with their GP (sample B). To preserve the anonymity of interviewees and to avoid the potential delineation of interviews (eg, by their order), we assigned a random number (from A1/B1 to A10/B10) to each interview [46].

### Attitudes and Expectations Toward Telemedicine

#### Usefulness of VC

In general, participants linked the use of VCs to perceived benefits. Although participants from sample A focused on 3 specific, positive aspects, participants from sample B mentioned several more factors they considered useful.

Of the 10 participants from sample A, 8 assumed that VC could be useful in saving their trip to the physician’s practice, as did the majority of interviewees from sample B. Participants associated the travel-saving effect of VC use with further benefits, that is, saving time and not being exposed to potential sources of infection from other patients:

Via Skype or the like, I would be able to talk to my doctor, tell him my problems. And if he could solve my problems right away, I wouldn’t have to go to the practice. That would be something I appreciate.

Interview A6

Participants from sample B found further aspects of VC beneficial, including higher flexibility to integrate an appointment into their daily routine and the prompt setting of a virtual appointment as opposed to an office appointment. Participants from sample B especially emphasized its practicality with regard to their own professional or informal obligations:

Well, concerning organization, it was quite easy, and of course quite practical, because I hadn’t to leave work. I had my appointment at 10 am, I just went into another room, where I was undisturbed. That’s just very comfortable.

Interview B9

Furthermore, half of all participants from sample B mentioned that a video appointment appeared to be more focused because of its transparent time limit. When using a web-based application form to receive an appointment for VC, participants were able to choose between different time slots, each comprising 10 min. Therefore, some interviewees argued that the scope of a specific appointment appeared to be clearer and more narrowed through digitization, as the timeframes of the appointments were displayed by the program they used to connect with their physician. In addition, they distinguished between appointments where their physical presence was necessary and those where their digital presence was sufficient. Overall, participants from sample B differentiated the usefulness of telemedicine systems to a higher degree and acknowledged more perceived benefits from digital appointments than did participants from sample A.

#### Security Aspects

Although interviewees were asked about the potential and actual disadvantages of VC, participants from both samples emphasized the need for data security. Participants were generally aware of the sensitivity of their medical data and expressed their concerns about the possibility of misuse. Foremost, interviewees described their personal medical data as vulnerable and transparent:

I already said it, the past shows how little you can trust the whole thing. I am as transparent as [...] this window.

Interview A7

[...] Technology certainly has, the definition of it certainly is to support humans and to be helpful, but every coin has got two sides, therefore every technology used by bad people holds the possibility to be misused.

Interview B8

Although the majority of interviewees from both samples A and B considered data security an important issue and a fundamental precondition for fully trusting a telemedicine system, participants from sample B put such statements into another perspective by stating that they risked the possibility of data insecurity to enjoy the benefits of VC:

But I don’t necessarily look at it that way, you might say, well data security, but I’m not attaching too much value on such things. See, we’ve so much data to disclose every day, you just have to be alert.

Interview B4

Well, it’s [digital appointment] working with video, internet, whatever. Who knows if it’s been recorded or what. In the beginning, I thought that way, but in the end, it’s nonsense. If it happens, it happens.

Interview B8

Accordingly, interviewees were aware of the importance of personal data in relation to the use of digital appointments. Participants who actually used VC compared the possibility of a breach of data with the normality of the potential misuse of data they could experience in comparable situations. In the end, the threat of data interception by third parties did not seem to outweigh the perceived advantages of digital appointments.

#### Operability of VC

As an antecedent to using digital technology, participants discussed the benefits of a preferably easy operation of a VC system. Although interviewees from sample A talked about prospective barriers they might have to face to use VC, participants from sample B emphasized the actual operability of the system they used for digital appointments:

My wife, she had to work with computers. Nowadays, she’s just like me, overstrained. Because she didn’t use it anymore.

Interview A8

Well, it was really easy. When you’re booking an appointment online, you have to register. Afterwards you just choose a time slot and you get an e-mail with a PIN, and within the e-mail there’s a link. And you even didn’t need to enter the PIN.

Interview B3

In addition, participants from sample B discussed possible features for extending the operability or functionality of the system they had experienced, such as the simultaneous transfer of personal medical data they collected by themselves (eg, blood pressure or coagulation level), better feedback functions while waiting for the physician to join the digital appointment, and an app to use instead of a website.

### Human Interaction and Its Impact on the Use of VC

#### Human Contact

Participants emphasized their need for personal and direct interactions. Although participants from both samples mentioned their concerns about technological changes leading to the replacement of direct physical contact between them and their physician, only participants from sample A expressed their wish for personal assistance regarding the use of VC at home. Overall, interviewees from sample A used the uniqueness of direct, personal human interaction as an argument to reject VC, whereas interviewees from sample B described situations in which they considered adequate digital appointments.

Nonetheless, for participants from both samples, personal contact with their physician remained highly important. Participants from sample A insisted on office visits and tended to exclude the possibility of audiovisual treatment from their own scope of action. Of the 10 participants from sample A, 8 mentioned the importance of a personal relationship with their physician, even if that meant accepting specific disadvantages. Similarly, participants from sample B also emphasized their need for office appointments as well:

Even if you have to wait a long time, the personal contact, you have to keep it upright.

Interview A1

It [video consultation] won’t work for every situation, logically. You need a personal talk. You need that.

Interview B4

Participants from sample A clearly distinguished between a physical meeting with their physician and contact mediated by VC. They seemed to assume that through personal contact, physicians are able to provide them with better care. VC was seen to restrict the senses of the physician and therefore limit the scope of examining a patient:

I don’t want that; I like to have personal contact. I think just from the way a patient behaves, as a doctor you’re able to recognize certain things [...] that cannot be transmitted through video.

Interview A5

In contrast, participants from sample B often assessed the appropriateness of a digital appointment through their actual interaction with their physician. They clearly perceived the specific limitations of a digital appointment, for example, the inability of their physician to examine them physically, to discuss severe diagnostic results, or deal appropriately in situations of high emotional stress:

A digital appointment, it’s limited by definition. You can’t, like when you’re actually in your physician’s practice, get a sonography, for example.

Interview B6

[...] when you get a bad diagnosis in a hospital and have to discuss it with your physician. When it’s really serious, and you like to talk about it, I’d rather do it face to face.

Interview B1

When I’m mentally unstable [...] when I face specific problems, I prefer to speak with someone in person. It’s maybe, I don’t know, it’s a matter of trust […].

Interview B4

Overall, participants from sample B differentiated the occasions for the use of telemedicine, whereas participants from sample A expressed their concern regarding a potential lack of physical and personal contact with their physician. Therefore, participants from sample B were able to provide specific situations that they preferred not to be digitally mediated.

#### Trust in Physician

Regarding the acceptance of digital appointments, participants from both samples discussed the relationship between them and their physician as a determining factor. Although even skeptical participants from sample A agreed to use VC when they were told to do so by their physician, interviewees from sample B emphasized the importance of trusting their physician to find the best medical solution for their problem, even without being physically present:

If my doctor says “Hey look, I’ve got a cool thing here, we’re able to communicate regularly. I am always there for you. If anything happens, you come to my practice, otherwise let’s try it that way,” I think if he says it that way, if the doctor I trust means it, it’s more likely I’ll do it.

Interview A3

And I think there has to be a trusting relationship to your doctor. To really want to test it [video appointment], to try something new, and to have trust in your physician, that everything will be ok, when you’re treated via video talk.

Interview B3

Although the role of the physician as a mediator between technology and the patient seemed to be essential to all participants, most interviewees from sample B indicated that nonetheless, some medical indications might justify digital treatment from an unfamiliar physician. Without being explicitly asked about it, some participants came up with the idea of being treated by unknown physicians for minor physical complaints (eg, a cold), a discussion of objective medical data (eg, test results), or highly urgent and acute symptoms (eg, an emergency):

When it’s just about a cold, or a cough, or whatever, it doesn’t really matter who’s treating me. As long as I’ve got the feeling of being taken seriously to some degree.

Interview B5

In summary, a trusting relationship between participants and physicians fostered a positive attitude toward the use of VC and might be considered an important condition for effective digital treatment. Furthermore, interviewees from sample B appeared to be partially willing to receive care from unfamiliar professionals to receive the perceived benefits from digital appointments.

### Rights and Obligations

#### Voluntariness of Use

Participants from both samples liked the idea of video appointments being an optional extension of the already existing primary care services and emphasized that using it needed to be a voluntary choice. Participants from sample B in particular recognized that choosing between a digital or an office treatment involved a bilateral process of negotiation between them and their physician:

It would be nice if my doctor doesn’t tell me to use it, but if he makes me an offer with specific advantages.

Interview A2

I think, I would appreciate having a voice. It’s one thing to say, well, when my doctor asks me “could we talk about it digitally?” [...] But you have to have a choice to say “no, I’d like to speak to you in person.”

Interview B5

Although participants would clearly like to choose a specific type of medical service voluntarily, interviewees also realized that their health status sometimes indicated a specific kind of medical service (digital or office) and agreed to follow the advice of their physician:

If you’ve got minor questions, concerning your medication or high blood pressure or anything else. Then you don’t have to come here, just get such a long distance consultation.

Interview A4

[...] and some appointments can be digitalized, you might ask your patient, what can be done digitally and when do you need an actual [office] appointment.

Interview B4

In general, participants from both samples seemed to express their wish to participate in the decision-making process regarding whether a digital appointment appeared to be adequate in a specific situation. Acknowledging the primary care physician’s professional assessment of their health status and indication for a specific service (digital or office treatment), participants emphasized the importance of the voluntary use of VC.

#### Availability of Care and VC

Participants from both samples were concerned about a present or future shortcoming of medical service in general because of a lack of professionals. Interviewees gave examples of long waiting times to get office appointments and severe problems in reaching their physician’s medical assistants via telephone:

Nobody answers the phone, when you’ve got something important to tell. Nobody’s answering it.

Interview A1

When I look at it, well, members of my own family were diagnosed with cancer tentatively, they needed an MRI really quick. They had to wait six months, every day they died of worry.

Interview B8

Broaching the issue of VC, participants from both samples generally described digital appointments as an opportunity to increase reachability and shorten waiting time:

In the morning I asked myself if I should go to the [physician’s] practice. Then I remembered he’s offering that service [digital appointment]. I logged in and had a look at it. When I had a closer look, I realized there was a slot vacant at 11 am. Wouldn’t have got a real [office] appointment that quick.

Interview B4

Although participants from sample B emphasized the benefit of digital appointments in increasing the availability of medical services and as an opportunity for primary care physicians to increase the number of patients they are able to care for, participants discussed potential disadvantages from their physician’s perspective, for example, an increased workload and unnecessary appointments because of the simplicity and availability of digital appointments. However, overall, participants from sample B suggested that VC might be able to counter the present challenges regarding the provision of care, which were mentioned by nearly all participants from both samples.

#### Patient Responsibilities

Only participants from sample B discussed the matter of self-responsibility regarding digital appointments. They mentioned that their own technological competence fostered the smooth processing of a digital appointment and that their own preparations were necessary beforehand:

Someday you’ll use it [video consultation] the first time and then you may realize that the camera won’t work or something. Surely, patients have to prepare. I’ve got the feeling, such an [digital] appointment, I have to write it in my calendar, it’s easy to forget, rather than actually going to the practice.

Interview B10

Necessary preparations were not reduced to technological issues. Participants mentioned that they had to focus on a specific issue rather than portray their pathogenetic history extensively. Furthermore, participants from sample B emphasized the importance of the competence to interpret one’s own symptoms and decide on an office or digital appointment:

Well, you’ve got a certain period of time, and when I’ve got my appointment, I know I can’t tell the whole story around it, for a quarter of an hour, but there are specific things [...]

Interview B9

But I think everyone’s able to judge, depending on your symptoms or pre-existing conditions, whether or not you have to go to the physician’s practice or if it’s suitable to use digital appointments.

Interview B3

In this regard, participants suggested that patients should carefully assess their health status, potential issues, and appropriate ways of dealing with them. Overall, interviewees from sample B reflected on the conditions for a satisfactory use of VC regarding their own possibilities of shaping an interaction between themselves and their physician.

### Social Factors

In general, several social factors influencing the use of technology can be found in our data. Unconsidered habitual attitudes toward digital technologies were often expressed in nonspecific, generic terms. Responses from both samples can be divided into statements concerning the social expectations of technology use in general and individual, private social interaction related to one’s own experiences with VC.

Interestingly, the majority of interviewees from sample A tended to express their readiness in a more passive way, assuring that they would not stand in the way of technological innovation, whereas participants from sample B stated their willingness to actively promote VC as an innovative technology. To explain user-specific readiness to use, participants from both samples often draw on stereotypes related to age:

So, I believe the willingness of older people to learn something new isn’t there. If I want to deal with it [new technologies], I have to be competent. Otherwise, when a problem occurs, something won’t work, and when the problem occurs a second time, they just throw it away. That’s how I see it.

Interview A6

Well, my mother, she was born in 1943, she’ll have trouble using it [video consultation], because she doesn’t know how to handle a pc, how to use a video chat function.

Interview B8

Participants from both samples reported the importance of talking to family members, friends, and colleagues about VC. Participants from sample A related their statements to relatively close family members and described their behavior as reactive, whereas interviewees from sample B considered themselves as being one of the first among their peers to use such innovative technology:

They always try to motivate us. “Daddy do this, do that,” they know I always decline, but their father complies with it.

Interview A4

Well, when I talk about it [use of video consultation] with my former wife, I have to add, we’ve got a good connection [...] she said, she’ll give it a try, because you’re just more flexible when you’re an employed person.

Interview B9

Overall, participants from sample B appeared to see themselves as pioneers when using VC. They actively discussed their experiences of digital appointments with peers and seemed to influence others rather than be influenced. Nonetheless, social interaction and the impact of social expectations and norms, including stereotypes, remain an essential factor in the use of technology.

## Discussion

The results shed light on factors that influence the attitudes, acceptance, and behavior of patients regarding the application of VCs as a representative of telemedicine in rural primary care. Studying preusers and actual users of telemedical solutions enables the empirical comparison of these 2 populations. The main findings are discussed against the background of technology design, application, and theory, thus delivering implications for practitioners, developers, and researchers.

### Differences in the Perception of Benefits and Security Issues

With regard to the participants’ expectations and perceptions toward the application of telemedicine in primary care, they showed high levels of perceived usefulness and beneficial effects of the technology. The literature on technology acceptance and adoption behavior has a vast corpus of studies that incorporate the perceived usefulness (TAM) and expected performance (UTAUT) of a technology as an antecedent to its use, together with associated intentions and attitudes [29]. A recent meta-analysis of research on the acceptance of consumer health technologies has shown that perceived usefulness can explain use behavior on a significant level [32]. Our study delivers further insights by considering both preusers and actual users of VC. As our findings indicate, preusers seem to attach less importance to the potential benefits of VC while focusing on other considerations for and against VC. Therefore, the inclusion of the patient’s role (preuser vs actual user) as a factor within research models on the acceptance of VC in primary care appears promising.

The preuser group mentioned only a few benefits they could think of, such as avoiding long and repeated travel to the practice. In contrast, the actual user group cited more examples of profitable outcomes. They experienced VCs to be more focused, efficient, and flexible. Literature has shown that there is no significant difference between text-based, information technology–mediated consultations and FTFC with regard to effectiveness as perceived by patients [50]. Our findings complement prior research on the use of VC in primary care, which deemed VC as a more thorough and convenient treatment method compared with FTFC [40,41,51] and telephone consultations [39], and indicate that video-based consultations are perceived as more effective and targeted than FTFC. Interestingly, while perceiving VC as a thorough approach [41,52], patients comply with the time limits of concise video meetings. Despite the limited time given, patients are satisfied with the experienced VC. From a practical standpoint, this makes it easier for GPs to schedule and adhere to appointments. On the contrary, preusers lack the experience of VC being a sufficient and satisfactory way of treatment. Here, the temporal limitation of virtual sessions can hinder patients from opting for VC. As research shows, patients are oftentimes skeptical about their health issues being addressed via VC depending on their condition, which renders VC inapplicable in certain situations [39,40,51].

Accordingly, from a practical standpoint, to increase the acceptance and use intentions of preusers, telemedical solutions such as VC systems should be promoted in more detail, clarifying what a VC can and cannot accomplish. It can be assumed that a higher awareness of benefits can lead to increased intentional and proactive use. In this regard, the benefits of VC have become apparent during the ongoing COVID-19 pandemic, which has put restrictions on the physical contact between GPs and their patients [53]. In times where access to care is limited, the potential of VC to bridge spatial gaps between GPs and patients has led to an uptake in VC implementation and use [54]. This is particularly true in rural areas that often lack comprehensive access to care [7]. Telemedicine, and VC in particular, enables GPs and clinicians to cope with given restrictions, maintain care of infected patients as well as those not related to COVID-19, and decrease infection rates [54].

As research shows [39], although the operability, usability, and ease of use of VC as well as the process of familiarizing oneself with the system are important to both user groups, the prevalence of security concerns and associated behavior varies. Although research on VC in primary care has focused primarily on the patient’s security in the sense of reducing physical harm and achieving health progress, our findings represent a novel aspect that contrasts preusers and actual users of VC. The preuser group indicates great concerns regarding the security of telemedicine and the potential of data misuse and leakage. In addition, preusers tend to affiliate these concerns with the intention of not using telemedicine. Actual users, while still aware of security issues, seem to be more willing to take risks in light of experienced benefits and convenience. The actual use of and exposure to telemedicine seems to alleviate patients’ concerns regarding technology-associated security. Literature has shown that the perceived benefits of technology use can outweigh perceived risks [55]. Accordingly, technology design should focus on alleviating the risks and threats perceived by preusers. From a design standpoint, to increase patients’ trust in telemedicine, technologies should present their privacy policies in an accessible and understandable way [56]. It appears to be important to incorporate ways of displaying technical security measures to the patient while not requiring high levels of technical skills, for instance, in the form of protection-ensuring labels [57] or a lucid and manageable list of people and institutions having access to the data [58]. This information can also be delivered to preuser patients by GPs to alleviate potential concerns that might not be perpetuated once the VC is experienced.

### Impacts of VC on the Patient-Physician Relationship

In the context of human interaction and its impact on the use of VC, the results indicate the importance of maintaining physical contact with the physician. The preuser group in our study expects fewer positive outcomes for virtual treatments and tends to reject the technology because in-office treatment by the physician is perceived to be superior. This finding is in line with prior studies on VC primary care, which indicate that the lack of physical contact potentially impedes adequate examination and proper treatment [59,60]. VC was deemed useful in nonurgent or routine situations [51]. In addition to this occasion-based opting for VC or FTFC, as our findings show, several patients requested aid by a competent person (eg, medical staff or peers) in case they had to use VC. This finding closely relates to the facilitating conditions that form an antecedent of the intention to use as well as the actual use of a technology in the UTAUT model. In particular, the model states that the degree of guidance and support experienced by the user when opting for a technology has an effect on their willingness to (continuously) use it [24,29]. Concerning our findings, this relation seems to be particularly relevant when dealing with preusers of VC in primary care. In the meantime, actual users seem to be able to fathom the feasibility and applicability of VC, enabling them to identify occasions and health issues that a digital treatment can address while placing less importance on guidance or external support. Apparently, patients are more able to differentiate occasions for office or digital treatments once they have conducted a VC with the physician. This finding concurs with studies that have shown that patients gain deeper knowledge about the occasions that are suitable for VC in comparison with FTFC when actively using the system [51].

Closely related to the relationship between patients and physicians, participants from both samples indicated that trust in the respective physician and an existing relationship are major drivers of technology acceptance and willingness to use it. Looking at investigations on technology acceptance and adoption behavior, trust in the opposite party (here, GPs offering VC) and their actions represents an important factor in the users’ technology assessment [32]. The findings indicate that a trusting patient-physician relationship increases the belief in an effective, beneficial, and safe treatment via VC, which is in line with prior studies on VC in primary care [39]. In the case of preusers, the data suggest that even obligatory technology use becomes more acceptable once interpersonal trust is achieved. Although actual users of VC have concrete experiences and specific benefits at their disposal, preusers tend to use trust as a heuristic input to decision making, making it easier for them to form an attitude [61]. Accordingly, the physician’s proactive invitation to arrange a digital appointment can potentially achieve higher use intentions once the relationship is considered trustworthy. By offering VC to the patient as an alternative to FTFC, the GP conveys that the virtual treatment is deemed suitable and beneficial, which could mitigate a patient’s potential concerns.

Revealing another interesting finding that complements the literature on VC in primary care, our study suggests that patients are partially willing to be treated via VC by a physician who is not their regular GP. Apparently, there are health-related occasions that go beyond the choice between VC and FTFC, which has been subject to prior studies [40,62-64] and further subdivide the feasibility of VC based on the need for trust. Both our findings and the literature show that there are suitable issues that can be addressed via VC but that call for different degrees of trust in the treating physician, such as receiving a severe diagnosis. There is still ambiguity on whether patients prefer a comforting environment (eg, at home) or an FTFC when talking about issues that are perceived to be sensitive or serious [60]. However, our findings reveal that there are health issues (such as a cold) that do not call for an already existing relationship with the physician. Accordingly, bringing together such patients with GPs who offer VC and are available for consultation represents a promising treatment model that further alleviates disparities in access to care. This concept can increase the number of patients who are suitable for treatment via VC while reducing the workload for the GPs responsible. This is particularly relevant in today’s health care because patients who can be treated virtually represent only a fraction of the clinical workload [63]. Therefore, based on the patient’s indication, perceived severity, and need for a trustworthy relationship, connecting patients with available physicians other than their own GP via VC promises a flexible and cost-effective way of delivering treatment [65].

### Emerging Tasks and Freedoms for Patients in a Virtual Setting

Looking at the patients’ rights and obligations that come along with the introduction of VC in primary care, the results show emerging freedoms, tasks, and behavioral patterns that patients should be aware of. Both samples wished for a voluntary and autonomous use of VC that enables them to adopt or reject the technology without disadvantages. The literature on technology acceptance has already identified the degree of voluntariness when choosing a technology as an important factor that influences users in their decision making [28,29]. Further research in the domain of health care technologies identified the patients’ freedom and preferences when choosing between VC and FTFC as an important factor that fosters their adoption or rejection of VC [40,51,59,60]. Our findings complement these studies by shedding light on the scenario in which using VC in primary care becomes obligatory and free of alternatives, for instance, in remote areas with detrimental access to care or in times of viral outbreaks such as the COVID-19 pandemic. Although the preuser group mentioned that they were willing to use an obligatory VC system if their physician suggested it, the actual user group indicated that they would obey telemedical obligations if they deemed the treatment occasion appropriate. That is, once a patient experiences VC and is able to fathom its applicability, the need for freedom of choice seems to decrease. Instead, actual users of VC tend to agree with obligatory digital appointments because they have gained the know-how regarding the way VC is applied in primary care. Theoretically speaking, they might have achieved higher levels of health literacy, which enables them to assess and understand health issues and necessary treatments [66] and computer self-efficacy, making them more competent in adequately choosing and using VC [67]. This is in line with previous research indicating that illiteracy with regard to proper technology use is a barrier to opting for VC instead of FTFC [68].

In addition to the degree of voluntariness in the use of VC, digital primary care also comes with obligations for the patient. Looking at prior research on the use of VC in primary care, our findings complement the first insights on the patient’s role in achieving an effective and satisfactory experience and use of technology. One of the first studies on VC in primary care indicated that patients perceive “[…] that they had responsibilities in ensuring the VC happened in an appropriate way, for example, conducting the VC in an appropriate setting […]” [39]. Further research raised the need for patients to prepare for a VC session, for example, by finding a private room and using headphones to secure privacy, as a novel consideration that is unique to telehealth [60]. Our data complement these findings and suggest that patients become aware of their roles and responsibilities through the actual use of the technology. Although the first sample did not mention this issue, the actual user group described the need to assess the feasibility of digital treatment as opposed to a physical visit. The participants stressed that prevalent health issues and potential treatments should be considered before making an appointment. When the participants considered a treatment via VC inappropriate for solving the health issues, they emphasized that a patient should be able to reject a digital appointment. Again, this requires patients to achieve higher levels of health literacy, so they are able to understand their condition, possible treatments, and the potential of telemedicine. Physicians might actually need to increase the effort of patient empowerment to ensure a degree of health literacy, which enables patients to decide what kind of treatment is appropriate in a specific situation [69,70]. With regard to technology design, the VC system can provide information about potentially prevalent diseases, feasible treatments, and contacts to specialized care to increase the patients’ health literacy and degree of empowerment. This information and potentially resulting measures by the patient can also be used to inform upcoming VCs, enriching patient-physician communication and mutual understanding.

### Social Impact on the Use and Design of VC

The data show different views on social factors in using VC. Apparently, the preuser group incorporated social cues and external norms into their attitude toward VC. The data suggest a subconscious trend toward social conformity when talking about technology in primary care. Interestingly, both groups gave credence to social stereotypes, claiming telemedicine to be more appropriate for younger generations. Preusers therefore seem to act according to what they think is the social norm, as suggested by prior studies on technology acceptance behavior [29,71,72]. In contrast, actual users talk about their influence on their peers. They appear (to themselves) to be innovative pioneers and inform their social cues about their mostly positive experiences. This is closely related to the image of the user (which the UTAUT model incorporates) coined as “[…] the degree to which use of an innovation is perceived to enhance one’s image or status in one’s social system” [29]. Although our findings show no support for actual users intentionally seeking to improve their image, their positive influence on their peers’ assessment of VC for treatment can still be identified. Thus, the patients’ self-perception as the first adopter of VC within their social system holds the potential to further explain why patients opt for VC in primary care and stick with it.

In addition, prior research on the use of VC in primary care has already shown that specific patient groups, such as older adults and the housebound, are perceived by GPs as not having the degree of technical skill to use VC effectively, although they would benefit from it the most [38]. Interestingly, the demographics of patients who opt for VC and those who do not differ significantly [68], indicating a social bias in the form of stereotyping [73]. Our study enhances these findings by indicating that lack of skill is also perceived among patients. As a result, to profit from the social dissemination of VC and its benefits, the resolution of these perceived gaps between patient groups by practitioners and policy makers seems necessary. GPs, for instance, are potentially able to achieve mutual understanding between patients and thus increase the intention to use VC by being transparent about the actual use of VC by different populations, including older adults. Furthermore, identified *pioneers* of VC can serve the GP as gatekeepers who influence their peers in a positive way.

At the design level, incorporating social cues and the adoption behavior of peers into telemedicine, and VC in particular, can potentially increase a patient’s willingness to (continuously) use it. Preusers, in particular, seem to highly value opinions and assessments by their peers. With regard to actual users, research shows that experienced users of virtual consultation increasingly form negative attitudes toward the use of the system [51]. From a theoretical standpoint, establishing and maintaining the use of VC can be achieved by finding ways to present behaviors of others to the patient, following the concept of *nudging* [74]. The idea of nudging is to gently encourage people to behave in a certain way at a subconscious level [75]. Nudges in the form of messages presented to the patient (eg, “Most of your friends have used VC before to contact their physician.”) can potentially lead to higher use intentions. Our findings expand prior research that shows that digital nudges can positively influence the willingness to use novel technologies in hospitals [76]. In turn, our findings contribute to the theoretical concept of nudging by indicating that the use of social norms as a nudging option [74] holds the potential to increase the acceptance rate of VC in primary care.

### Limitations

This study has some limitations. First, the sampling procedure is prone to selection bias because we did not strictly regulate participant characteristics and demographics. Thus, the sample yields varying degrees of technical affinity and age, which could frame the results in a certain direction. People opting for telemedicine (representing sample B) might exhibit particular characteristics such as dispositional innovativeness that could partially explain patient perceptions and behavior. In addition, the interviews were partially conducted during the COVID-19 pandemic, which could have influenced the responses of the participants. As VC is the only way for many patients to consult their GP, at least during the acute times of the pandemic, interviewees might have formed stronger intentions and more positive reactions to the technology. Second, it is difficult to discuss identified factors in comparison with patients living in urban areas because the data are limited to the chosen context. The urban patients’ experiences of VC and their intention to participate might differ with regard to the varying structural circumstances and quantity of practitioners. Third, participants were recruited in a limited region. Nevertheless, this area is representative of rural regions in Germany according to size and demographic characteristics. Further studies should be conducted to shed light on urban environments and enable rural-urban comparisons in a reliable and insightful way.

### Conclusions and Outlook

This study investigates factors that constitute patients’ attitudes, perceptions, and technology acceptance behavior regarding the use of VC in the rural primary care setting. To account for different levels of experience with technology use, this study involves the perspectives of preusers as well as actual users of VC. The empirical data enable the comparison of these 2 perspectives and the provision of implications for the design, application, and theory of VC. The study delivers an in-depth description and discussion of patients’ experiences and attitudes that complement our understanding of the use of VC in primary care by involving both preusers and actual users of VC. The findings can be of interest to researchers, medical practitioners, and designers of VC and telemedicine solutions, further enabling them to increase the behavioral intentions of preusers, maintain continuous use of VC by already experienced patients, and achieve a critical mass of patients participating in digital treatments.

With regard to the patients’ behavioral intentions toward and actual use of VC in primary care, that is, their technology acceptance behavior, this study unveils several links to established models and includes antecedents of health care technology acceptance. Interestingly, when looking at models that have been put up and tested by researchers to investigate patients’ acceptance of consumer health technologies, none of these models (TAM, TPB, or UTAUT) combines the factors of perceived usefulness, trust in GP, social norms and image, degree of voluntariness and obligatory use, patient responsibility and involvement, and need for physical contact, which our findings suggest [32]. Hence, proposing and testing a theoretical model that integrates these antecedents represents a promising avenue for technology acceptance researchers when investigating the use and acceptance of VC in primary care. In addition, the comparison of user groups shows that the priorities, needs, expectations, and attitudes toward using VC in primary care vary between preusers and actual users. Therefore, the inclusion of both patient groups appears to be feasible when testing new theoretical models of technology acceptance by patients. The role of the patient (preuser vs actual user) thus holds potential explanatory power when looking at antecedents of core constructs such as intention to use VC.

This paper opens up many further research opportunities for future work as well as for preceding studies. First, research can be conducted to further investigate the gap between different generations regarding their perceptions and opinions on telemedicine. The findings suggest that stereotyping takes place across all ages, that is, the association of older adults with a lack of technical skills or the perceived social pressure coming from younger generations. Second, to overcome the monomethod approach, studies engaging wider and more heterogeneous populations can be conducted, for instance, in the form of surveys conducted on the web or on the GP’s site. In doing this, researchers can gather data with higher external validity and achieve further insights into how to implement digital technologies within the primary care setting, based on quantitative measures. Here, interventional studies appear to be feasible to shed light on the behavioral and attitudinal changes triggered by the use of digital technology. Third, to generate feasible and beneficial designs for technology, the involvement of technology experts and developers, working together in focus groups and workshops, can yield concrete technical features and innovations that further improve the comprehensive provision of primary care in rural areas.

## Appendix

Multimedia Appendix 1 Sample and interview characteristics.

Multimedia Appendix 2 Interview guidelines.

Multimedia Appendix 3 Code quantities.

## Abbreviations

FTFC: face-to-face consultation
GP: general practitioner
TAM: Technology Acceptance Model
TPB: Theory of Planned Behavior
UTAUT: Unified Theory of Acceptance and Use of Technology
VC: video consultation
