# Supplementary material for: Investigating the Acceptance of Video Consultation by Patients in Rural Primary Care: Empirical Comparison of Preusers and Actual Users
Source: JMIR Med Inform. 2020 Oct 22;8(10):e20813. doi: 10.2196/20813 (PMC7644376; doi:10.2196/20813)
Supplement: Multimedia Appendix 1 [file medinform_v8i10e20813_app1.doc]

**Multimedia Appendix 1**

The table below illustrates the sample and interview process characteristics. Note that two of the interviews in sample A each engaged a married couple, leading to a total of 10 interviews but 12 interviewees.

|  | Characteristic | Sample A (Pre-users) | Sample B (Actual users) | Total |
| --- | --- | --- | --- | --- |
|  |  |  |  |  |
| **Participants** |  |  |  |  |
|  | N | 12 | 10 | 22 |
|  | Age (range) | 30 - 81 years | 23 - 69 years | 23 - 81 years |
|  | Age (mean) | 59 years | 41 years | 51 years |
|  | Gender | 5 females, 7 males | 4 females, 6 males | 9 females, 13 males |
|  | Patient type | 7 acute, 5 chronic | 4 acute, 6 chronic | 11 acute, 11 chronic |
| **Interviews** |  |  |  |  |
|  | N | 10 | 10 | 20 |
|  | Time | August 2019 | February to April 2020 |  |
|  | Duration (range) | 17 - 37 min. | 19 - 47 min. | 17 - 47 min. |
|  | Duration (mean) | 22 min. | 30 min. | 26 min. |
|  | Sampling method | Convenient | Purposeful |  |
