# Supplementary material for: Investigating the Acceptance of Video Consultation by Patients in Rural Primary Care: Empirical Comparison of Preusers and Actual Users
Source: JMIR Med Inform. 2020 Oct 22;8(10):e20813. doi: 10.2196/20813 (PMC7644376; doi:10.2196/20813)
Supplement: Multimedia Appendix 2 [file medinform_v8i10e20813_app2.doc]

**Multimedia Appendix 2**

**Interview guideline used for interviewing sample A**

General: Information about the interviewee

- Age and gender
- Professional situation and education
- Family status

1. Patient factors

- Do you see a doctor regularly or in a fixed cycle?
- Do you think that you can assess your health status and perform any necessary measures by yourself?
- Have you ever encountered digital technologies in a health context?
- How do you assess your skills in dealing with digital technologies?

2. Social factors

- What do family/friends think about novel digital technologies?
- How do others’ experiences and opinions influence your own view/behaviour?
- Are you being guided by others when using digital technologies?

3. Environmental factors

- How do you assess the availability of medical care services in your area?
- How is your connection to your doctor’s practice? (Travel, duration, waiting time)
- Does this influence your evaluation of technologies being used in health care?

4. Organizational factors

- How would you describe the relationship with your general practitioner? (Differentiation between personal and digital contact, satisfaction, influence on evaluation of technology)

5. Technical and interaction factors

- How do you assess the usefulness of digital technologies in healthcare, in general and personally?
- What advantages and disadvantages do you see in the application of technologies?
- Would you like to decide for yourself if and which technologies to use? What if you are "forced" to use it?

Closing

- How do you envisage future medical care, in general and personally?

**Interview guideline used for interviewing sample B**

General: Information about the interviewee

- Age and gender
- Professional situation and education
- Family status

1. Patient factors

- Do you visit your family doctor’s practice regularly?
- How would you rate your skills in dealing with digital technologies and the video consultation in particular?
- Did you have any concerns or expectations before the first use?
- Can you describe your experience when using it the first time?
- What were the significant changes or innovations for you?

2. Social factors

- What do your family/friends think about telemedicine such as the video consultation?
- How do others’ experiences and opinions influence your own view/behaviour?
- Are you being guided by others when using digital technologies and the video consultation in particular?

3. Environmental factors

- How do you assess the availability of medical care services in your area?
- How do you describe the accessibility of your medical practice? (Travel, duration, waiting time)
- What influence does the video consultation have on this in your perception?

4. Organizational factors

- How would you describe the relationship with your general practitioner? (Differentiation between personal and digital contact, satisfaction, influence on evaluation of technology)
- Would you say that the video consultation has influenced this relationship?
- How do you evaluate the digital treatment? (quality of treatment and procedure)
- Do you notice differences between in-office and digital treatment?

5. Technical and interaction factors

- How do you assess the usefulness of the video consultation, in general and personally?
- What advantages and disadvantages do you see in the use of telemedicine?
- What is your motivation to use it? Are there barriers?
- Would you like to decide for yourself if and when you use telemedicine?
- In which situations do you prefer in-office over digital treatment?
- In your opinion, what does the long-term establishment of telemedicine such as the video consultation depend on?

6. Adjacent telemedical scenario

- Can you imagine that other digital components are used for diagnostics and treatment besides the experienced video consultation? (e.g., mobile sensors, which record data and send it to the doctor)
- Do you think such components are useful? If yes, in which situations?

Closing

- How do you envisage future medical care, in general and personally?
