# Supplementary material for: Investigating the Acceptance of Video Consultation by Patients in Rural Primary Care: Empirical Comparison of Preusers and Actual Users
Source: JMIR Med Inform. 2020 Oct 22;8(10):e20813. doi: 10.2196/20813 (PMC7644376; doi:10.2196/20813)
Supplement: Multimedia Appendix 3 [file medinform_v8i10e20813_app3.doc]

**Multimedia Appendix 3**

The following table depicts the quantities of selective codes (i.e., factors) that have been assigned to the data during the re-coding step. In addition, it is shown in how many interviews the respective factor occurred. Since we want to compare pre- and actual users of video consultations, the statistical data for both samples and in total are presented. Sample A yielded 260 and sample B 353 codes, which leads to a total of 613 codes.

| Factors | Sample | Codes | Interviews |
| --- | --- | --- | --- |
|  |  |  |  |
| **Human Contact** |  |  |  |
|  | Sample A | 33 (12 %) | 9 (90 %) |
|  | Sample B | 45 (12 %) | 10 (100 %) |
|  | Total | 78 (12 %) | 19 (95 %) |
| **Social Factors** |  |  |  |
|  | Sample A | 39 (15 %) | 9 (90 %) |
|  | Sample B | 34 (9 %) | 10 (100 %) |
|  | Total | 73 (11 %) | 19 (95 %) |
| **Usefulness of Video Consult.** |  |  |  |
|  | Sample A | 40 (15 %) | 10 (100 %) |
|  | Sample B | 69 (19 %) | 10 (100 %) |
|  | Total | 109 (17 %) | 20 (100 %) |
| **Security Aspects** |  |  |  |
|  | Sample A | 17 (6 %) | 7 (70 %) |
|  | Sample B | 17 (4 %) | 7 (70 %) |
|  | Total | 34 (5 %) | 14 (70 %) |
| **Voluntariness of Use** |  |  |  |
|  | Sample A | 25 (9 %) | 8 (80 %) |
|  | Sample B | 17 (4 %) | 10 (100 %) |
|  | Total | 42 (6 %) | 18 (90 %) |
| **Availability** |  |  |  |
|  | Sample A | 54 (20 %) | 10 (100 %) |
|  | Sample B | 60 (16 %) | 10 (100 %) |
|  | Total | 114 (18 %) | 20 (100 %) |
| **Trust in Physician** |  |  |  |
|  | Sample A | 16 (6 %) | 7 (70 %) |
|  | Sample B | 42 (11 %) | 10 (100 %) |
|  | Total | 58 (9 %) | 17 (85 %) |
| **Operability of Video Consult.** |  |  |  |
|  | Sample A | 36 (13 %) | 10 (100 %) |
|  | Sample B | 48 (13 %) | 10 (100 %) |
|  | Total | 84 (13 %) | 20 (100 %) |
| **Patient Responsibilities** |  |  |  |
|  | Sample A | 0 (%) | 0 (0 %) |
|  | Sample B | 21 (5 %) | 10 (100 %) |
|  | Total | 21 (3 %) | 10 (50 %) |
